# Serum glucose and risk of cancer: a meta-analysis

**DOI:** 10.1186/1471-2407-14-985

**Published:** 2014-12-19

**Authors:** Danielle J Crawley, Lars Holmberg, Jennifer C Melvin, Massimo Loda, Simon Chowdhury, Sarah M Rudman, Mieke Van Hemelrijck

**Affiliations:** King’s College London, School of Medicine, Division of Cancer Studies, Cancer Epidemiology Group, London, UK; Regional Cancer Centre, Uppsala-Örebro, Uppsala University Hospital, Uppsala, Sweden; Department of Surgical Sciences, Uppsala University, Uppsala, Sweden; Department of Pathology, Harvard Medical School, Boston, MA USA; Pathology, Dana-Farber Cancer Institute, Boston, MA USA; Department of Oncology, Guy’s & St Thomas’ NHS Foundation Trust, London, UK

**Keywords:** Glucose, Cancer, Metabolic syndrome, Meta-analysis, Diabetes

## Abstract

**Background:**

Raised serum glucose has been linked to increased risk of many solid cancers. We performed a meta-analysis to quantify and summarise the evidence for this link.

**Methods:**

Pubmed and Embase were reviewed, using search terms representing serum glucose and cancer. Inclusion and exclusion criteria focused on epidemiological studies with clear definitions of serum glucose levels, cancer type, as well as well-described statistical methods with sufficient data available. We used 6.1 mmol/L as the cut-off for high glucose, consistent with the WHO definition of metabolic syndrome. Random effects analyses were performed to estimate the pooled relative risk (RR).

**Results:**

Nineteen studies were included in the primary analysis, which showed a pooled RR of 1.32 (95% CI: 1.20 – 1.45). Including only those individuals with fasting glucose measurements did not have a large effect on the pooled RR (1.32 (95% CI: 1.11-1.57). A stratified analysis showed a pooled RR of 1.34 (95% CI: 1.02-1.77) for hormonally driven cancer and 1.21 (95% CI: 1.09-1.36) for cancers thought to be driven by Insulin Growth Factor-1.

**Conclusion:**

A positive association between serum glucose and risk of cancer was found. The underlying biological mechanisms remain to be elucidated but our subgroup analyses suggest that the insulin- IGF-1 axis does not fully explain the association. These findings are of public health importance as measures to reduce serum glucose via lifestyle and dietary changes could be implemented in the context of cancer mortality.

## Background

Diabetes mellitus is a risk factor for many chronic diseases including cardiovascular disease and cancer. People with diabetes are 2-fold more likely to die from cancer than those without [1]. Therefore, it is thought that pre-diagnostic elevated blood glucose levels are associated with risk of cancer [2–4]. Several epidemiological studies have investigated this association. The largest being a Korean cohort study of over one million men and women found a hazard ratio for all solid cancers of 1.22 (95% CI: 1.16-1.27) for men in the fifth quintile compared to the first quintile [5].

Despite the growing evidence for an association between diabetes and carcinogenesis [6], the mechanism by which raised glucose contributes to risk of cancer is not fully established [7]. The insulin – insulin growth factor (IGF)-1 axis is a commonly suggested pathway. It is thought that insulin resistance, which impairs the action of insulin and occurs in individuals with type 2 diabetes or metabolic syndrome, leads to prolonged hyperinsulinaemia. This decreases the production of IGF-binding proteins, which consequently results in raised IGF-1 levels and cellular changes leading to carcinogenesis via increased mitosis and reduced apoptosis [8]. It is, however, important to note that hyperinsulinemia during the early stages of diabetes may play a role in carcinogenesis independent of IGF-1 [9].

Another suggested pathway between glucose and risk of cancer is the reduced hepatic production of sex hormone binding globulin (SHBG) following prolonged hyperinsulinaemia [8]. This leads to an increase of available sex hormones, such as oestrogen and testosterone, which can drive carcinogenesis in hormonal sensitive cancers like postmenopausal breast or prostate cancers [8].

Elevated glucose can result in a state of chronic inflammation which changes the cytokine micro-environment and leads to an increase of cytokines such as interleukin 6 (IL-6) [10], tissue necrosis factor alpha (TNF-α) [11, 12] and vascular endothelial growth factor (VEGF) [13]. These changes can lead to an increase in tumour cell motility, invasion and even tumour metastasis [14, 15].

Finally, glucose may have a direct role in cancer development as it is a key nutrient. It is needed for proliferating cells and several types of tumour cells have been shown to have up-regulated glucose transporters [16].

Given the above-suggested pathways and the increasing prevalence of diabetes and cancer, this meta-analysis aims to summarize and quantify the existing evidence for a link between raised serum glucose and risk of all solid cancers. Using data from epidemiological studies on adult participants whose serum glucose levels and cancer diagnoses were assessed, this study aims to answer the question whether there is a higher risk of solid cancer in those with raised glucose levels, compared to those with normal levels.

## Methods

This meta-analysis was conducted following the PRISMA statement for completing systematic reviews and meta-analyses [17].

### Literature search strategy

A computerised literature search of databases (Pubmed search followed by an Embase search) to identify full text and abstracts published within the last fifteen years, which included only adult human subjects was performed. “Grey literature” such as abstracts, letters, articles presented at relevant conferences and meetings, was also reviewed. The search was done with and without MESH terms (serum glucose, blood glucose, cancer, neoplasm). We also conducted cancer-specific searches for prostate, breast, colorectal, oesophageal, gastric, pancreatic, liver, lung, ovarian, endometrial, cervical, testicular, bladder, melanoma, brain, thyroid and head and neck cancers. All references of the selected articles were checked, including hand searches.

The final articles were chosen based on the following set of inclusion criteria: the publication pertained to an epidemiological study which measured circulating serum levels of glucose (fasting or non fasting); the reference level of high glucose was clearly defined; risk of a non-fatal solid cancer (any type) was assessed as an outcome; the analytical methods were well-described with sufficient and relevant data available; predominantly non-diseased adult study populations were used; a minimum of 20 cases were included. Studies measuring glucose only after an oral glucose load were excluded. The literature review and data collection was conducted by DC and reviewed by MVH.

**Figure 1:**
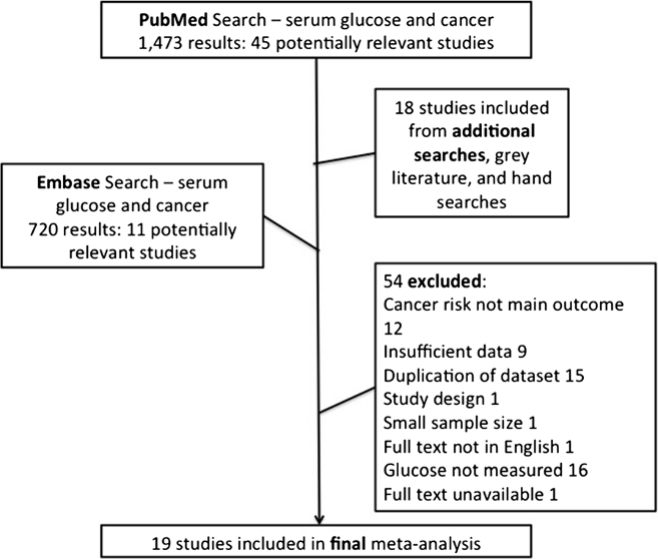
**Flow chart of study selection.**

Initially, titles were reviewed to assess whether they met inclusion criteria. Titles that indicated the study met these criteria progressed to an abstract review. Upon inclusion after this step, the full manuscript was thoroughly checked to evaluate inclusion and exclusion criteria. Additional studies were considered from grey literature and hand searches (N = 18). Unpublished data on glucose and risk of breast, prostate, and colorectal cancer was also obtained from the MECAN group, allowing us to use this large dataset in the analysis of all cancers [18]. Figure 1 provides more detailed information regarding the exclusion process. More specifically, 12 studies were excluded because incident cancer risk was not the main outcome of interest [17, 19–29], ten studies did not provide the data to calculate number of cases with high and normal glucose levels [4, 30–38], 13 studies were using data which was already used in another included publication [39–51], one study was cross-sectional and addressed correlations instead of risks [52], one study included less than 20 cases [53], one was not published in English [54], 16 did not provide data on serum glucose levels prior to cancer diagnosis [33, 55–70], and one study was not available through our different data resources [71].

The following details were recorded for each study: author, year of publication, country where study was undertaken, sex of participants, age range, type of cancer, type of study, fasting or non fasting glucose measurements and number of cases and total subjects for each glucose range. To allow for comparison all values in conventional units (mg/dl) were converted into SI units (mmol/L) [72].

### Statistical methods

The association between serum glucose and cancer risk was evaluated by calculating the pooled relative risk (RR) with a random effects model to allow for possible heterogeneity between studies. A cut-off of > 6.1 mmol/L was used to define high glucose, consistent with values used in WHO definition of metabolic syndrome [73]. The included studies all used different cut off points for glucose levels, some used tertiles, others quartiles or quintiles. For the sake of this analysis all data was dichotomised into ‘high’ and ‘normal’ as close to the 6.1 mmol/L cut off as possible by combining groups above and below this level.

An initial meta-analysis was performed using all studies. Potential heterogeneity was assessed with weighted forest plots, which display the relative risk estimate of cancer depending on glucose level. Potential publication bias was assessed with a contour enhanced funnel plot, as well as Beggs Test [41, 42]. We also performed stratified analyses by study type and sex. We then conducted cancer-specific analyses for prostate, breast, and colorectal cancer, as these were the most commonly investigated cancers. We also conducted a secondary analysis excluding those studies which did not specify the fasting status of the glucose samples. Given the suggested complex aetiology between diabetes, glucose, and cancer, we additionally conducted stratified analyses based on potential underlying mechanisms – below referred to as hormone-driven and IGF-1-driven [74, 75]. Although the identification of which cancers are driven by the IGF-1 axis, is not entirely elucidated, the cancers for which the most consistent supporting evidence is available are prostate, colorectal and breast cancer [9, 52, 75, 76]. Hence, here we considered these as ‘IGF-1 driven’ cancers. Breast, endometrial and prostate cancers were also combined for a separate subgroup of ‘hormone driven’ cancers [23, 30, 55]. All analyses were performed on STATA version 12.0.

## Results

The Pubmed search resulted in a total of 1,473 studies, 45 of which were deemed as initially relevant. A further 11 were identified via an Embase search and 18 from hand searches and grey literature, resulting in a total of 74 potentially relevant papers. Using the above-defined criteria, 55 were excluded (Figure 1).

A total of 19 studies were included in the primary analysis: six cohort, six case cohort, three hospital-based case–control, and four nested case–control studies. Nine studies were conducted in Europe, seven in Asia and three in the USA. Three studies presented data on all solid cancers, five on colorectal cancer, four on prostate cancer, two on breast cancer, two on endometrial cancer and one paper each for pancreatic, renal and hepatocellular cancers (Table 1).

**Table 1:** **Summary of study characteristics included in primary analysis**

Author/Year	Country	Sex	Cancer (s) included	Timing of measurement glucose	Method for glucose assessment	Study type	Cases	Age range	Adjusted for	Main finding
Yun et al. 2012 [77]	Korea	Male	Prostate	Fasting	Hitachi 7600 automatic chemical analyser using hexokinase method	Case control#	166	66.4 (mean)	Age, BMI	OR for 2^nd^ and 3^rd^ tertile compared to 1^st^ tertile: 1.63 (95% CI: 0.92-2.88) and 1.70 (95% CI: 0.91-3.18)
Albanes et al. 2009 [78]	Finland	Male	Prostate	Fasting	Hitachi 912 Chemistry Analyzer using the hexokinase reagent	Case cohort	100	50-69	Age, BMI	OR for 2^nd^, 3^rd^, and 4^th^ quartile compared to 1^st^ quartile: 1.33 (95% CI: 0.72-2.48), 0.95 (95% CI: 0.46-1.86), and 1.43 (95% CI: 0.76-2.68)
Chung et al. 2006 [79]	Korea	Both	Colorectal	Fasting	Enzymatic colorimetric test	Case control#	105	35-75	Age, sex, BMI, triglycerides, cholesterol	OR for 2^nd^ and 3^rd^ tertile compared to 1^st^ tertile: 2.0 (95% CI: 0.9-4.4) and 3.0 (95% CI: 0.9-9.8)
Jee et al. 2005 Male [5]	Korea	Male	All Cancers	Fasting	Not specified	Cohort	37759	45.3 (mean)	Age, age squared, amount of smoking, alcohol use	HR for 2^nd^, 3^rd^, 4^th^, and 5^th^ quintile compared to 1^st^ quintile: 1.01 (95% CI: 0.99-1.04), 1.13 (95% CI: 1.09-1.17), 1.16 (95% CI: 1.08-1.24), and 1.22 (95% CI: 1.16-1.27)
Jee et al. 2005 Female [5]	Korea	Female	All Cancers	Fasting	Not specified	Cohort	16074	49.6 (mean)	Age, age squared, amount of smoking, alcohol use	HR for 2^nd^, 3^rd^, 4^th^, and 5^th^ quintile compared to 1^st^ quintile: 1.02 (95% CI: 0.99-1.06), 1.03 (95% CI: 0.96-1.10), 1.03 (95% CI: 0.93-1.13), and 1.15 (95% CI: 1.01-1.25)
Hsing et al. 2003 [80]	China	Male	Prostate	Fasting	Radioimmunoassay with sensitivity limit of 0.5 ng/mL	Case control*	128	N/S	Age, total calories, BMI	OR for 2^nd^ and 3^rd^ tertile compared to 1^st^ quartile: 0.81 (95% CI: 0.46-1.44), and 1.68 (95% CI: 1.01-2.80)
Wulaningsih et al. 2013 [81]	Sweden	Both	All Cancers	Non specified	Enzymatically with a glucose oxidase/peroxidase method	Cohort	1021	20>	Age, gender, socioeconomic status, fasting,co-morbidities	HR: 1.08 (95% CI: 1.02-1.14) per standardized log of glucose
Cust et al. 2007 [82]	Western Europe	Female	Endometrial	Non specified	Enzymatic colorimetric test	Case control*	284	59.9 (mean)	Study centre, menopausal status, age, time of day of blood collection, fasting status, phase of menstrual cycle (pre menopausal)	OR for 2^nd^, 3^rd^, and 4^th^ quartile compared to 1^st^ quartile: 1.01 (95% CI: 0.58-1.74), 1.59 (95% CI: 0.89-2.83), and 1.62 (95% CI: 0.89-2.95)
Limburg et al. 2006 [83]	Finland	Male	Colorectal	Fasting	Hitachi 912 Chemistry Analyzer using the hexokinase reagent	Case cohort	134	50-69	Smoking pack years, BMI, protein intake, fat intake, fibre intake, alcohol consumption, caloric intake, history of DM, occupational physical activity	HR for 2^nd^, 3^rd^, and 4^th^ quartile compared to 1^st^ quartile: 1.19 (95% CI: 0.58-2.43), 1.95 (95% CI: 0.97-3.91), and 1.65 (95% CI: 0.78-3.49)
Stolzenberg-Solomon et al. 2005 [84]	Finland	Male	Pancreatic	Fasting	Assay performed on a chemical analyser	Case cohort	169	52-69	Age, years of smoking and BMI	HR for 2^nd^, 3^rd^, and 4^th^ quartile compared to 1^st^ quartile: 1.15 (95% CI: 0.66-2.02), 1.49 (95% CI: 0.86-2.59), and 1.69 (95% CI: 0.97-2.94)
Yamada 1998 et al. [85]	Japan	Both	Colorectal	Fasting	Enzymatically using commercially available kits	Case control*	129	34-73	Age, sex, BMI, smoking, alcohol consumption	OR for 2^nd^, 3^rd^, and 4^th^ quartile compared to 1^st^ quartile: 1.0 (95% CI: 0.6-1.7), 0.7 (95% CI: 0.3-1.5), and 2.0 (0.9-4.4)
Schoen et al. 1999 [86]	USA	Both	Colorectal	Fasting		Cohort	102	65 >	Age, sex, physical activity	
Zhang et al. 2010 [87]	China	Female	Endometrial	Fasting	Abbott Aeroset TM fully Automatic Biochemical Analyzer	Case control#	942	N/A	Menopausal status, BMI	OR: 4.34 (95% CI: 3.48-5.42) for high versus low serum glucose levels
Gunter et al. 2009 [88]	USA	Female	Breast	Fasting	Assay with sensitivity of 0.5 mg/dL	Case cohort	835	50-79	Age, race, alcohol consumption, smoking, FHx breast cancer, parity,age at menarche, age at first childs birth,use of OCP, NSAIDs, HRT, educational attainment, endogenous estrodiol levels, BMI, physical activity	HR for 2^nd^, 3^rd^, and 4^th^ quantile compared to 1^st^ quantile: 1.14 (95% CI: 0.82-1.59), 0.99 (95% CI: 0.70-1.38), and 0.92 (95% CI: 0.65-1.29)
Sieri et al. 2012 [89]	Italy	Female	Breast	Fasting	Enzymatic UV test using a fully automated system with sensitivity of 0.04 mmol/L	Case control*	356	35-69	Age, education, age at first birth, age at menarche, parity, FHx breast cancer, OCP, breastfeeding, alcohol intake, smoking	OR for 2^nd^, 3^rd^, and 4^th^ quartile compared to 1^st^ quartile: 1.18 (95% CI: 0.84-1.66), 1.29 (95% CI: 0.89-1.86), and 1.63 (95% CI: 1.14-2.32)
Gunter et al. 2008 [44]	USA	Female	Colorectal	Fasting	Assay with sensitivity of 0.5 mg/dL	Case cohort	438	50-79	Age	HR for 2^nd^, 3^rd^, and 4^th^ quantile compared to 1^st^ quantile: 0.94 (95% CI: 0.66-1.34), 0.91 (95% CI: 0.63-1.30), and 1.16 (95% CI: 0.83-1.63)
Van Hemelrijck et al. 2011 [90]	Sweden	Both	Renal	Mixed	Enzymatically with a glucose-oxidaseperoxidase method	Cohort	958	55.4 (mean)	Age, gender, creatinine, triglycerides, total cholesterol, fasting status, SES	HR for 2^nd^, 3^rd^, and 4^th^ quartile compared to 1^st^ quartile: 0.97 (95% CI: 0.77-1.21), 1.09 (95% CI: 0.88-1.35), and 1.19 (95% CI: 0.97-1.46)
Van Hemelrijck et al. 2011 [91]	Sweden	Male	Prostate	Mixed	Enzymatically with a glucose-oxidaseperoxidase method	Cohort	5112	20-80	Fasting status,triglyceride and total cholesterol quartile , SES, time btw measurement and cohort entry	HR for 2^nd^, 3^rd^, and 4^th^ quartile compared to 1^st^ quartile: 0.93 (95% CI: 0.86-1.01), 0.93 (95% CI: 0.85-1.01), and 0.87 (95% CI: 0.81-0.94)
Chao et al. 2011 [92]	China	Male	Liver	Fasting	Automatic dry-chemical analyzer	Case cohort	124	30-65	Age, smoking, alcohol consumption, FHx of HCC, HBV viral load, HCV genotype ,HbeAg status, BCP double mutations	HR for 2^nd^ and 3^rd^ tertile compared to 1^st^ tertile: 1.40 (95% CI: 0.80-2.45) and 2.37 (95% CI: 1.12-5.04)
Stocks et al. 2009 [18]	Western Europe	Male	All Cancers	Mixed	Mixture of non-enzymatic, serum/enzymatic, and plasma/enzymatic	Cohort	18621	44.7 (mean)	Age, BMI, smoking status	HR for 2^nd^, 3^rd^, 4^th^, and 5^th^ quintile compared to 1^st^ quintile: 1.07 (95% CI: 0.90-1.25), 1.10 (95% CI: 0.93-1.29), 1.18 (95% CI: 1.02-1.37), and 1.18 (95% CI: 1.00-1.37)
Stocks et al. 2009 [18]	Western Europe	Female	All Cancers	Mixed		Cohort	11664	45 (mean)	Age, BMI, smoking status	HR for 2^nd^, 3^rd^, 4^th^, and 5^th^ quintile compared to 1^st^ quintile: 0.87 (95% CI: 0.70-1.07), 0.90 (95% CI: 0.73-1.10), 1.18 (95% CI: 0.97-1.42), and 1.29 (95% CI: 1.07-1.59)

*Nested case–control studies; #Hospital-based case–control studies.

**Figure 2:**
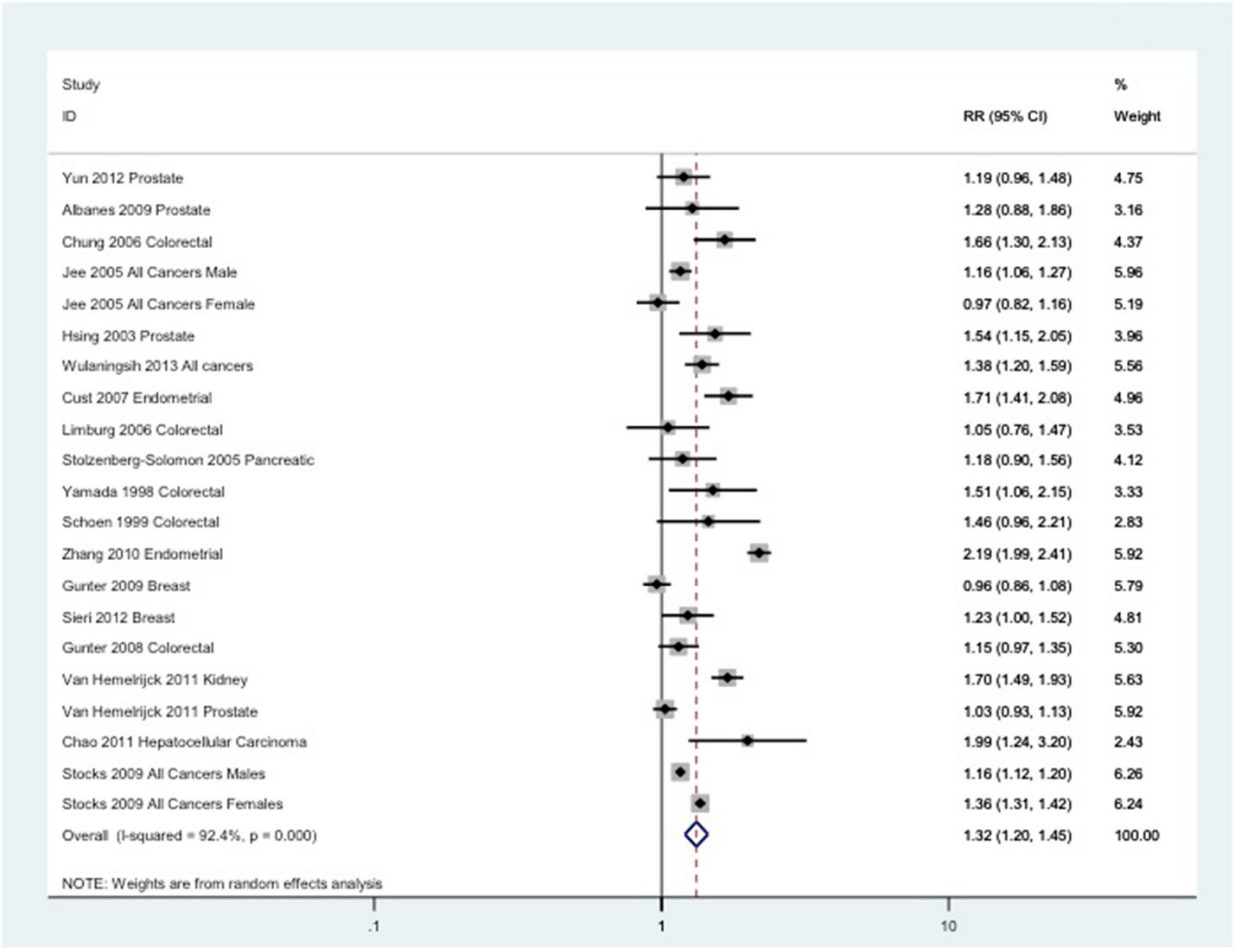
**Forest plot for studies comparing risk of cancer by serum glucose levels with serum glucose < 6.11 mmol/L as the reference category.**

The random effects analysis comparing overall cancer risk by serum glucose levels showed a pooled relative risk (RR) of 1.32 (95% CI: 1.20 – 1.45) for high versus normal levels of serum glucose (Figure 2). The I^2^ statistic showed heterogeneity (I^2^ = 92%; P < 0.05), even though every individual estimate indicated a positive association. Hence, we conducted a ‘remove one’ analysis to gauge each study’s impact; the I^2^ statistic did not fall below 85%. Next, we conducted a sensitivity analysis using studies which included ‘all cancers’ as the outcome versus those with site specific outcomes. The heterogeneity remained high and the RR did not change drastically. When looking at “All cancers” as an outcome, the RR was 1.21(95% CI: 1.09-1.34) with and I^2^ of 92%. When combining all site-specific cancers as an outcome, the RR was 1.38 (95% CI: 1.16-1.63) with an I^2^ of 92%. Tumour-specific analyses were performed for the three most commonly studied cancers and resulted in pooled relative risks of 1.09 (95% CI: 0.95-1.25), 1.35 (95% CI: 1.21-1.51) and 1.14 (95% CI: 1.04-1.26), for breast, colorectal and prostate cancer, respectively. The related I^2^ statistic was 74% for breast, 57% for colorectal, and 53% for prostate.

**Figure 3:**
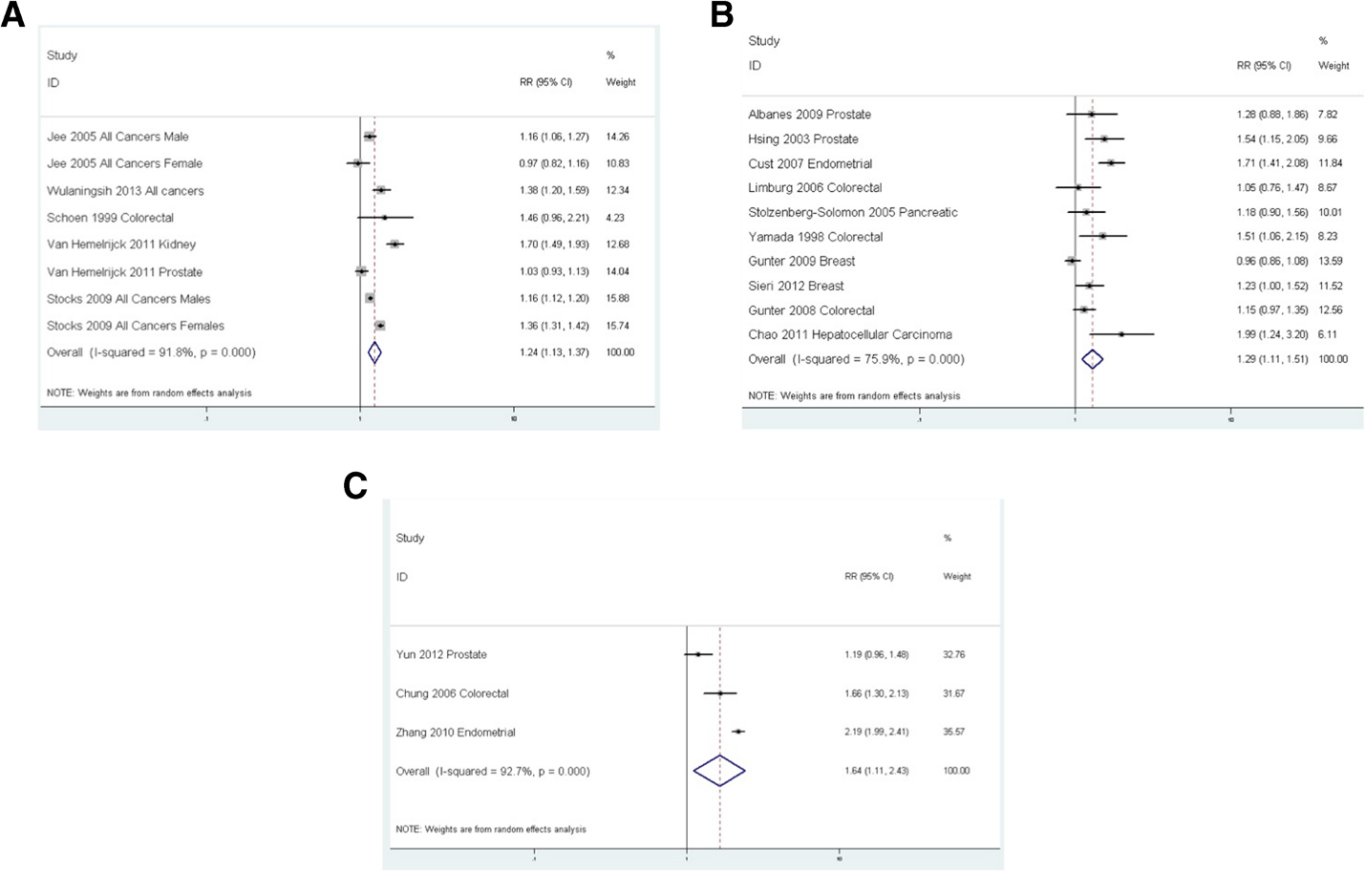
**Forest plots. a**: Forest plots for cohort studies comparing risk of cancer by serum glucose levels with serum glucose < 6.11 mmol/L as the reference category. **b**: Forest plots for nested case–control and case-cohort studies comparing risk of cancer by serum glucose levels with serum glucose < 6.11 mmol/L as the reference category. **c**: Forest plots for hospital-based case–control studies comparing risk of cancer by serum glucose levels with serum glucose < 6.11 mmol/L as the reference category.

**Figure 4:**
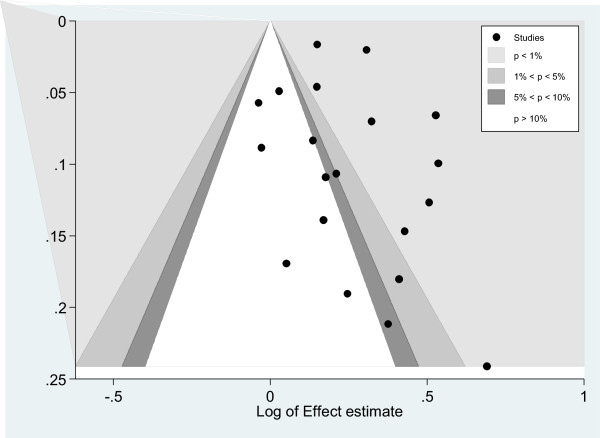
**Contour enhanced funnel plot for meta-analysis comparing risk of cancer by serum glucose levels with serum glucose < 6.11 mmol/L as the reference category.**

A stratified analysis by study type showed similar pooled RRs for cohort studies, case-cohort/nested case–control studies and hospital-based case–control studies (Figure 3): 1.24 (95% CI: 1.13-1.37), 1.29 (95% CI: 1.11-1.51), and 1.64 (95% CI: 1.11-2.43). The I^2^ statistic was 92%, 76%, and 93%, respectively (Figure 4).

The overall pooled RR was 1.17 (95% CI: 1.09-1.25) for men and 1.32 for women (95% CI: 1.06-1.63). Studies where it was not possible to stratify by sex showed a pooled RR of 1.55 (95% CI: 1.40-1.71). The I^2^ statistic for these sex-stratified analyses was 48% for men, 96%, for women and 19% where it was not possible to stratify by sex. Including only those with fasting glucose measurements did not have a large effect on the pooled RR either (RR: 1.32 (95% CI: 1.11-1.57). The I^2^ statistic was 92%.

The pooled RR for hormonally driven cancers was 1.34 (95% CI: 1.02-1.77; I^2^: 96%) versus 1.41 (95% CI: 1.20-1.66; I^2^: 69%) for the non-hormonally driven cancers. IGF-1-driven cancers showed a pooled RR of 1.21 (95% CI: 1.09-1.36; I^2^: 67%) versus 1.73 (95% CI: 1.40-2.12; I^2^: 85%) for those not thought to be driven by IGF.

When assessing publication bias, the funnel plot showed an area of missing studies which includes regions of both low and high statistical significance suggesting that both studies that showed a non-significantly and significantly inverse association between glucose and cancer were missing. Therefore, under the assumption that studies are being suppressed because of a mechanism based on two-sided p-values, publication bias cannot be accepted as the only cause of funnel asymmetry.

## Discussion

This is the first meta-analysis examining the association of serum glucose and cancer risk. We found a consistent positive association, which was not altered strongly by sex, study type, or cancer type.

As previously described, several molecular mechanisms have been postulated in an effort to explain the association between glucose and carcinogenesis. The insulin – IGF-1 axis is the most commonly suggested pathway [93]. Our results showed a weaker association for IGF-1 driven cancers than the overall association or non-hormonally driven cancers, suggesting that if the insulin- IGF- 1 axis does play a role it is likely to be as part of a more complex molecular mechanism.

Another proposed mechanism is an increased availability of sex hormones caused by a reduction of SHBG in the presence of hyperinsulinaemia [7, 94]. However, our meta-analysis showed a similar association between elevated serum glucose and risk of hormonally and non-hormonally driven cancers. This suggests that this is not the only underlying mechanism for the link between glucose and cancer. It is possible that other mechanisms, i.e. chronic inflammation [10–12] or direct actions of glucose [16], may also be playing a role.

To our knowledge this is the first comprehensive meta-analysis looking at epidemiological studies of serum glucose levels and cancer risk. Existing meta-analyses to date focused on the association between serum glucose levels and a specific type of cancer [4, 76]. A breast cancer-specific study including ten cohort studies found that the association between serum glucose levels and risk of breast cancer was small in non-diabetic subjects (pooled RR: 1.11 (95% CI: 0.98-1.25) [4]. The direction of this study is consistent with our findings, however our meta-analysis focused on high serum glucose levels as defined by the WHO definition for metabolic syndrome so that we also included potential diabetic subjects. Thus, when investigating serum levels of glucose, it is also important to consider diabetes. A bladder cancer-specific study showed that diabetes was associated with a 30% increased risk (95% CI: 1.18-1.43), which is consistent with the direction of the association found for serum glucose and cancer in our meta-analyses [76]. Other cancer types which also show a positive association with diabetes include pancreatic, endometrial, breast and colorectal cancer [20–22, 95], however an inverse association has been observed for prostate cancer [91]. The latter must be interpreted with caution as diabetics have higher morbidity and mortality from other diseases. There may be competing risks masking their risk of prostate cancer [96]. However, it is important to note that diabetes is a slightly different exposure than serum levels of glucose as diabetic treatments may normalise glucose levels and potentially also affect risk of cancer [43].

We made every effort to include all relevant publications available to date through various sources, including grey literature, and the two main online databases (Pubmed and Embase). We were able to also access unpublished data from the MECAN group enabling us to include this large cohort of over 500,000 subjects [18]. In addition, clearly defined objective criteria for exposure, outcome, and other study characteristics were specified *a priori*. One limitation of our study is the heterogeneity between the different categorization methods for glucose ranges across the include studies. We tried to overcome this by combining the different categories as similarly as possible and believe this cannot significantly affect our findings. Nevertheless, this made it not possible in the current meta-analysis to make a distinction between pre-diabetes and diabetes. The overall results showed a rather large amount of heterogeneity, as suggested by the I^2^ statistic. All of our sensitivity and subgroup analyses showed consistent findings in terms of direction of the association, while the heterogeneity remained high. Only when we conducted tumour specific analysis, the I^2^ statistic reduced. This suggests that heterogeneity is most likely explained by combining studies with different outcomes. However, the consistent finding of a positive association in all our analyses supports the robustness of our findings. Six of the studies included, either had mixed or did not specify fasting status. However, exclusion of these studies did not alter the association observed. A further limitation is the lack of information regarding the diagnosis of diabetes, use of oral hypoglycaemics or insulin in those included in the studies. Future research including adjustment for components such as age, cancer treatment, diabetes (or its treatments), or BMI would be useful in confirming the importance of raised glucose in carcinogenesis. All studies included were soundly designed and executed epidemiological studies, which clearly defined their methodology. However, the size of the studies did vary considerably. The two largest studies [5, 18] did account for well over half of the cases included, but they represent a Korean and European population which we believe can be broadly applicable to all patient populations. Limitations reported by the individual studies overlap widely. They include, having only localised cancer as an outcome, small sample size, specific demographic groups only (i.e. smokers only), lack of information on diabetes and obesity and all but one study [81] used single measurements of glucose for their analysis.

## Conclusions

A positive association was found between serum glucose levels and risk of cancer. The heterogeneity observed between studies calls for more translational studies investigating how serum glucose is associated with carcinogenesis. However, given there were seven million deaths from cancer worldwide in 2011 and it is estimated that more than a third were attributable to modifiable risk factors [97], these findings are of public health importance as measures to reduce serum glucose via lifestyle and dietary changes could be implemented to reduce risk of cancer.

## Abbreviations

BMI: Body mass index
95% CI: 95% confidence interval
IGF-1: Insulin growth factor −1
SHBG: Sex hormone binding globulin
IL-6: Interleukin 6
TNF-alpha: Tissue necrosis factor alpha
VEGF: Vascular endothelial growth factor
WHO: World Health Organisation
RR: Relative risk.
